# Glycolytic Proteins Interact With Intracellular Melatonin in *Saccharomyces cerevisiae*

**DOI:** 10.3389/fmicb.2019.02424

**Published:** 2019-10-24

**Authors:** María Ángeles Morcillo-Parra, María José Valera, Gemma Beltran, Albert Mas, María-Jesús Torija

**Affiliations:** Department de Bioquímica i Biotecnologia, Facultad d’Enologia, Universitat Rovira i Virgili, Tarragona, Spain

**Keywords:** melatonin, fermented beverages, glycolysis, GADPH, enolase, pyruvate kinase

## Abstract

Melatonin is a bioactive compound that is present in fermented beverages and synthesized by yeast during alcoholic fermentation. Many studies have shown that melatonin interacts with some mammalian proteins, such as sirtuins or orphan receptor family proteins. The aim of this study was to determine the intracellular synthesis profile of melatonin in *Saccharomyces cerevisiae* and to identify the proteins that may interact with this molecule in yeast cells. Melatonin from fermentation samples was analyzed by liquid chromatography mass spectrometry, and proteins bound to melatonin were immunopurified by melatonin-IgG-Dynabeads. Melatonin was produced intracellularly in the lag phase of yeast growth and was exported to the extracellular media during the stationary phase. During this period, melatonin was bound to six proteins with molecular weights from 55 to 35 kDa. Sequence analysis showed that most proteins shared high levels of homology with glycolytic enzymes. An RNA-binding protein was also identified, the elongation alpha factor, which is related to mitochondria. This study reports for the first time the interaction of melatonin and proteins inside yeast cells. These results highlight the possible role of melatonin as a signal molecule and provide a new perspective for understanding its role in yeast.

## Introduction

Melatonin (N-acetyl-5-methoxytryptamine) is an indole amine synthesized from L-tryptophan (Reiter, 1991) that presents antioxidant activity and has been associated with the regulation of circadian rhythm and reproduction in humans (Reiter et al., 2007; Serrano et al., 2010; Galano et al., 2011). Recently, melatonin has also been associated with a protective function against oxidative stress and UV radiation in *Saccharomyces* yeast (Vázquez et al., 2017, 2018; Bisquert et al., 2018).

Melatonin has been described in many organisms, including bacteria, algae, fungi, insects, and plants (Hardeland and Poeggeler, 2003). Several studies have also revealed the presence of melatonin in many fermented beverages, such as beer (Kocadağlı et al., 2014), fermented orange beverages (Fernández-Pachón et al., 2014), and wine (Mercolini et al., 2008; Stege et al., 2010; Rodriguez-Naranjo et al., 2011; Vitalini et al., 2013; Wang et al., 2016; Fernández-Cruz et al., 2017, 2018). Although the occurrence of melatonin in fermented beverages is low (at pg–ng/mL levels), these concentrations have been demonstrated to contribute sufficiently to the dietetic intake to exhibit measurable effects (Hornedo-Ortega et al., 2016).

When the winemaking process is monitored, *S. cerevisiae* produces significant amounts of melatonin and other methoxyindoles during standard yeast growth and alcoholic fermentation, which highlights the role of yeast, particularly *Saccharomyces cerevisiae*, in the production of melatonin in wine. The concentration of melatonin reaches its maximum between the first and second day of fermentation (Sprenger et al., 1999; Arevalo-Villena et al., 2010; Rodriguez-Naranjo et al., 2012; Vigentini et al., 2015). Production also depends on precursor availability; tryptophan is essential as it is the principal precursor, and its presence increases and accelerates the synthesis of melatonin (Sprenger et al., 1999; Rodriguez-Naranjo et al., 2012). However, a recent study has detected melatonin produced from serotonin and 5-methoxytriptamine pulse (Muñiz-Calvo et al., 2019).

The different functions of melatonin in human cells suggest the existence of specific receptors. Many studies have associated melatonin with two transmembrane proteins that belong to the GPCR superfamily as the receptors of this molecule in the mammalian membrane (MT1 and MT2) (Reppert, 1997), which are encoded by *MTRN1A* and *MTNR1B* genes, respectively (Jockers et al., 2016).

In addition, melatonin has also been detected in cell nuclei. Previous studies with [^3^H] melatonin showed the existence of interaction sites for the binding of this molecule in the nuclei on orphan receptor family proteins (Becker-André et al., 1994; Carlberg and Wiesenberg, 1995; Wiesenberg et al., 1995). Thus, other nuclear proteins, such as calreticulin, a ubiquitous protein that is involved in intracellular signaling pathways under Ca^2+^ binding, have been associated with melatonin interactions (Macías et al., 2003). Calreticulin is multifunctional and may play an important role in the modulation of a variety of cellular processes.

On the other hand, melatonin has also been associated with sirtuins, class III histone deacetylase enzymes that regulate the cell cycle, DNA repair, cell survival, and apoptosis and have important roles in normal and cancer cells (Mayo et al., 2017). Sirtuin genes are highly conserved in organisms ranging from archaea to humans. In fact, the first sirtuin gene to be identified in yeast was the “silent information regulator 2” (*SIR2*) (Braunstein et al., 1993; Blander and Guarente, 2004), which is responsible for gene silencing at mating type loci, telomeres, or rDNA (Smith and Boeke, 1997). Nevertheless, only mammalian sirtuins have been associated with melatonin (Das, 2005).

Although several authors have demonstrated that melatonin is synthesized by yeast during alcoholic fermentation (Sprenger et al., 1999; Arevalo-Villena et al., 2010; Rodriguez-Naranjo et al., 2011, 2012; Vigentini et al., 2015; Fernández-Cruz et al., 2017, 2018; Muñiz-Calvo et al., 2019), its role inside yeast cells and what signals trigger its synthesis are unclear. The aim of this study was to determine the intracellular synthesis profile of melatonin in yeast during alcoholic fermentation and the fate of this molecule inside the cell before its excretion into the extracellular medium. To this end, we first analyzed the intracellular and extracellular melatonin levels produced by *S. cerevisiae* during fermentation conditions. We subsequently performed immunopurification melatonin-IgG-Dynabeads and identified a set of proteins that interact with melatonin inside the cell. This study reports for the first time the interaction of melatonin and proteins in yeast cells.

## Materials and Methods

### Yeast Strain and Inoculum Preparation

In this study, we used one strain of *S. cerevisiae,* QA23, from Lallemand S.A. (Canada). The yeast was rehydrated in water at 37°C for 30 min and plated on YPD plates [1% (w/v) yeast extract, 2% (w/v) glucose, 2% (w/v) bacteriological peptone, and 2% (w/v) agar (Panreac Quimica SLU, Barcelona, Spain)] for 48–72 h at 28°C. Afterward, the preculture was prepared in 50 mL of YPD broth [1% (w/v) yeast extract, 2% (w/v) glucose, and 2% (w/v) bacteriological peptone] and shaken overnight at 120 rpm and 28°C. Then, yeast cells were transferred into fresh minimal medium [1X yeast nitrogen base without amino acids or ammonia (Becton, Dickinson and Company, Sparks, MD, USA), 2% (w/v) glucose, and 350 mM (NH_4_)_2_ SO_4_ (Panreac Quimica SLU, Barcelona, Spain)] and cultured for 3 days at 28°C and 120 rpm.

### Alcoholic Fermentation Conditions

Synthetic grape must (SM) was prepared based on Beltran et al. (2004) with some modifications: the aromatic amino acid (tryptophan, tyrosine, and phenylalanine) concentration was increased five-fold in relation to the regular concentration (González et al., 2018). These increased concentrations of aromatic amino acids occurred at the expense of the remaining amino acids to maintain the concentration of YAN (yeast assimilable nitrogen) (300 mg/L). A total of 450 mL of medium, inoculated with 2 × 10^6^ cells/mL from the minimal medium culture, was placed in 500-mL bottles. Fermentations were performed in triplicate at 28°C with continuous orbital shaking (120 rpm). Cell populations were evaluated by measuring the optical density (OD_600nm_), and 10^8^ cells were collected at different time points during yeast growth. Samples were centrifuged at 12,000 rpm for 3 min at room temperature. The supernatant was stored at −20°C for extracellular melatonin analysis, and the pellet was washed with distilled water, frozen in liquid nitrogen, and stored at −80°C for intracellular melatonin analysis.

### Melatonin Analysis

Intracellular metabolites were extracted by adapting the boiling buffered ethanol method previously described by Gonzalez et al. (1997). Briefly, 1 mL of a boiling solution of 75% (v/v) absolute ethanol containing 70 mM HEPES buffer (pH 7.5) was added to the cell pellet (10^8^ cells). This mixture was incubated for 3 min at 80°C. The extract was concentrated by evaporation at 45°C in a SpeedBack (Concentrator *plus*, Eppendorf Ibérica, Madrid, Spain). The final intracellular content was resuspended in 1 mL of Milli-Q water and centrifuged for 10 min at 5,000 rpm to remove the insoluble particles. The supernatant was transferred to a new tube and stored at −20°C until use.

Intracellular and extracellular melatonin samples were extracted with chloroform. Briefly, 50 μL of sample was mixed with Milli-Q water (1:1, v:v). Then, 10 volumes of chloroform were added. Samples were shaken for 1 h at 1,200 rpm. The organic phase was dried under a flow of nitrogen gas and resuspended in 50 μL of a mixture of methanol and water (40:60, v:v). Then, the samples were centrifuged for 5 min at 14,500 rpm. Supernatants were transferred and analyzed.

Samples were analyzed by performing liquid chromatography mass spectrometry (LC-MS/MS) following the method described by Rodriguez-Naranjo et al. (2011) with some modifications. The system was based on a high-performance liquid chromatography coupled to a triple quadrupole mass spectrometer (Agilent G6490; Agilent Technologies, Palo Alto, CA, USA). Melatonin separation was performed using an Agilent 150 × 2.1 mm i.d., 3.5 μm, Zorbax Sb-18 column. Chromatographic separation was performed using a binary gradient consisting of (A) water and (B) methanol as LC grade solvents, both containing 0.1% (v/v) formic acid. The elution profile was 100% B (4 min) and 10% B (6 min). The analysis temperature was set at 40°C. The flow rate was 0.4 mL/min. The injection volume was 7 μL. Melatonin quantification was performed using Agilent MassHunter WorkStation Software Quantitative Analysis Version B0104 by comparing the 233/174 transition MS data of the sample and the standard. Samples were analyzed by triplicate, and the standard deviation was calculated.

### Protein Purification

Samples from different fermentation times were purified by a Pierce™ Crosslinking Magnetic IP/Co-IP Kit (Thermo Fisher Scientific, Waltham, MA, USA). Briefly, melatonin IgG-Dynabeads were prepared by crosslinking anti-melatonin rabbit IgG polyclonal antibody to Dynabeads (LifeSpan BioSciences, Seattle, WA, USA). Then, 10^8^ cells were resuspended in 1 mL of extraction buffer (25 mM Tris, 150 mM NaCl, 1 mM EDTA, and 1% NP40, pH 7.4) and lysed by glass beads by applying three shaking cycles of 1 min in Mini Beadbeater-24 (BioSpec Products, Bartlesville, OK, USA) and 1 min on ice. Lysed cells were centrifuged at 3,000 rpm and 4°C for 10 min to remove insoluble particles. Melatonin IgG-Dynabeads were added to each lysate, and the suspension was rotated for 1 h at room temperature. The melatonin IgG-Dynabeads were collected with a magnet and washed three times with 500 μL of ice-cold extraction buffer. Isolated proteins were eluted from the melatonin IgG-Dynabeads by following manufacture’s instructions and resolved by 12% SDS-PAGE. Proteins in the gel were visualized by Pierce™ Silver Stain kit (Thermo Fisher Scientific, Waltham, MA, USA). ImageJ software^1^ was used to quantify the protein intensities in the SDS-PAGE analysis.

### Protein Digestion and Analysis

Visible gel bands were excised from the silver-stained gel and washed with 25 mM ammonium bicarbonate (ABC) pH 8 and acetonitrile. Samples were reduced by adding 10 mM dithiothreitol for 1 h at 56°C and alquilated with 55 mM iodoacetamide for 30 min at room temperature in darkness. Then, the samples were washed with 25 mM ABC pH 8 and a mixture of 25 mM ABC and acetonitrile (50%, v:v). For digestion, gel bands were first rehydrated with sequencing-grade trypsin solution at a ratio of trypsin:protein 1:100 (w/w) (considering 50 μg of protein per band), covered with 50 mM ABC, and incubated overnight at 37°C. Extraction of peptides from gels was performed by incubating them with a mixture of acetonitrile (50%) and formic acid (5%) for 15 min and collecting the supernatant. The final extraction step was performed with acetonitrile (100%). Before mass spectrometry analysis, samples were desalted using HLB SPE (Waters, USA) and resuspended in 25 μL of 0.1% of formic acid.

Peptides were analyzed by nanoLC-(Orbitrap) MS/MS [LTQ-Orbitrap Velos Pro mass spectrometer (Thermo Fisher, San José, CA, USA)]. Two microliters of sample (~1 μg of protein digest) was separated into a C18 reversed-phase (RP) nanocolumn (75 μm i.d., 15 cm length; 3 μm particle diameter, NikkyoTechnos Co. LTD, Japan) coupled to a trap nanocolumn (100 μm i.d., 2 cm length, 5 μm particle diameter, Thermo Fisher Scientific) using a 60-min acetonitrile gradient (A = water, 0.1% formic acid; B = acetonitrile, 0.1% formic acid). The flow rate during the elution gradient was 300 nL/min. For real-time ionization and peptide fragmentation, an enhanced FT-resolution spectrum (resolution = 30,000 FHMW) was used, followed by a data-dependent IT-MS/MS scan from the most intense 10 parent ions with a charge state rejection of one using a CID fragmentation with a normalized collision energy of 35% and dynamic exclusion of 0.5 min.

### Protein Identification Analysis and Relative Quantification

Tandem mass spectra were extracted and charge states deconvoluted by Proteome Discoverer version 1.4.0.288 (Thermo Fisher Scientific, Waltham, MA, USA). All MS and MS/MS samples were analyzed using Mascot (Thermo Fisher Scientific; version 2.4.1.0). Mascot (v2.5) was set up to search SwissProt_2018_03.fasta database (557,012 entries), restricting for *S. cerevisiae* taxonomy (7,904 sequences) and for other mammals (13,162 sequences) and assuming trypsin digestion. Three missed cleavages and an error of 0.8 Da for fragment ion mass and 10 ppm for precursor ion were allowed. Oxidation of methionine and acetylation of the N-terminal were specified as variable modifications, whereas carbamidomethylation of cysteine was set as a static modification. The false discovery rate was set at 0.01. For proteins identified only with one peptide, visual verification of fragmentation spectra was performed.

## Results and Discussion

### Melatonin Production

Fermentations with the QA23 strain were performed using SM, which ensured the reproducibility of the experiment, that was enriched in aromatic amino acids (five-fold normal concentration) because yeast growth conditions can influence levels of methoxyindoles (Sprenger et al., 1999). Different time points during the fermentation process were analyzed.

**Figure 1:**
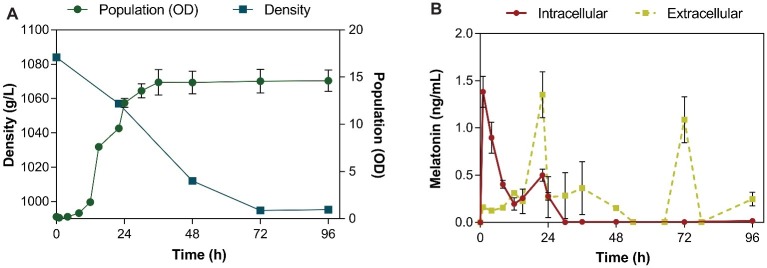
Fermentation kinetics of the QA23 yeast strain **(A)** by monitoring density (■) and population (●) throughout the fermentation. Intra- (●) and extracellular (■) concentrations of melatonin **(B)** during alcoholic fermentation performed by the QA23 *S. cerevisiae* strain. The intracellular concentration of melatonin is represented in ng/mL per 10^8^ cells.

In addition to melatonin content determination, fermentation parameters such as density and yeast growth were measured. QA23 completed the fermentation with no residual sugars (<1 g/L, Figure 1A) in 3–4 days, similar to results achieved in previous studies using the same conditions (Lleixà et al., 2016; González et al., 2018).

Intracellular melatonin was observed in the first hour of fermentation (lag phase, Figure 1B), reaching its maximum at 1.38 ng/mL per 10^8^ cells. After this first peak, the intracellular levels of melatonin rapidly decreased, until the late exponential phase, when another peak of melatonin concentration was detected at 22 h (0.5 ng/mL). The first part of the growth curve (0–6 h) is expected to be crucial for yeast as only essential molecules are formed, while the yeast is trying to adapt to the conditions of the new medium (Rodriguez-Naranjo et al., 2012).

In contrast, in the extracellular media, melatonin first appeared when yeast cells were in late exponential phase (22 h; Figure 1B), confirming previous results of other authors that reported the detection of extracellular melatonin at 24 h (Rodriguez-Naranjo et al., 2012; Vigentini et al., 2015; Valera et al., 2019). At this point, metabolism is very active to support exponential yeast growth. After that, yeast cells entered the stationary phase, and the melatonin concentration decreased. When all sugars were almost consumed, extracellular melatonin peaked again at 72 h (1.15 ng/mL; Figure 1B). A previous study reported that the maximum production of melatonin occurred at the end of alcoholic fermentation (Fernández-Cruz et al., 2018).

Although intracellular melatonin production occurred very quickly during the lag phase, probably due to the importance of some molecules in the yeast adaptation process to the new conditions (Rodriguez-Naranjo et al., 2012), the melatonin levels inside the cell rapidly decreased and could not be detected in the extracellular media until late exponential and stationary phase. Given the difference between intracellular production and extracellular detection of melatonin, a possible interaction with some molecules inside the cell, such as proteins, could be responsible for this gap in melatonin secretion. This interaction could be part of its transport to the extracellular medium or be related to a possible signaling function of this molecule during yeast growth.

### Protein Analysis

**Figure 2:**
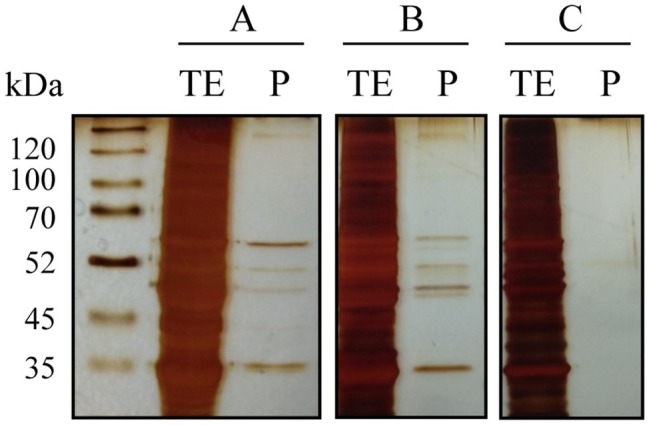
SDS-PAGE gel electrophoresis of total protein extracts before (TE) and after purification with melatonin IgG-Dynabeads (P) from three yeast samples (A–C). In all cases, the same total protein concentration was used. In samples A and B, several proteins bound to melatonin were observed, although with different intensities, while in sample C, no proteins were bound.

During alcoholic fermentation, several samples were collected to determine a possible interaction between melatonin and proteins. The proteins bound to melatonin IgG were purified from crude extract using a Pierce™ Crosslinking Magnetic IP/Co-IP Kit. After SDS-PAGE gel electrophoresis, proteins bound to the anti-melatonin antibody were excised and in-gel trypsin digested, and the resulting peptides were analyzed by nano LC-(Orbitrap) MS/MS. The protein purification protocol was confirmed by gel electrophoresis of the total protein extract (TE) and the purified proteins (P) (Figure 2). Several protein bands with molecular weights ranging from 55 to 35 kDa were bound specifically to melatonin.

**Figure 3:**
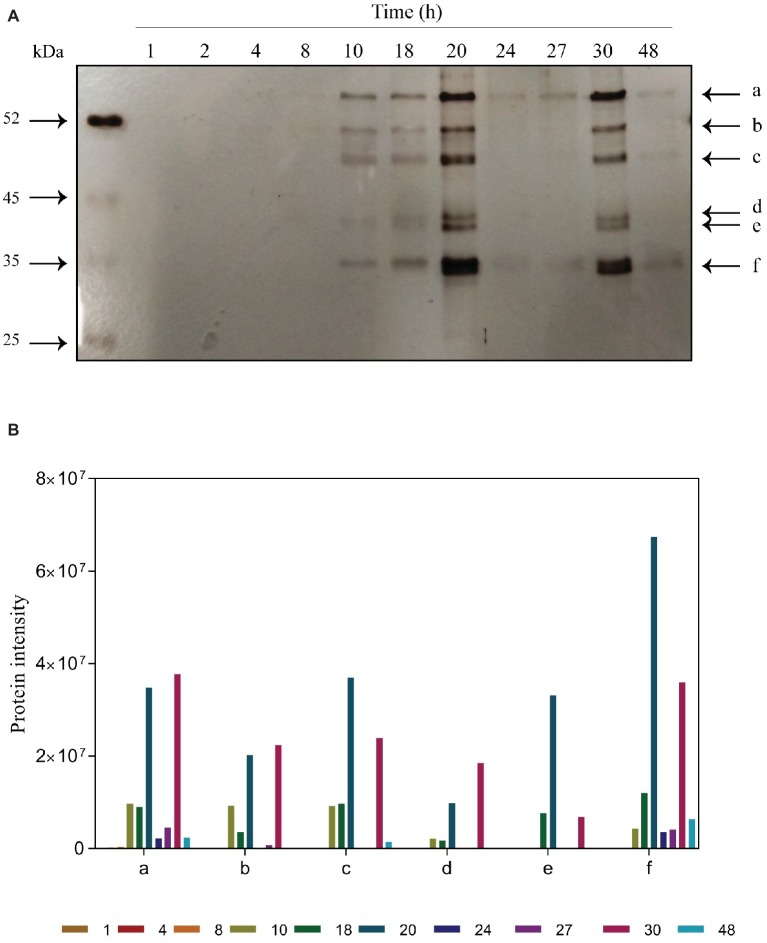
Time course of purified proteins during alcoholic fermentation. **(A)** SDS-PAGE gel electrophoresis. Bands marked are proteins bound to melatonin: a, pyruvate kinase 1; b, elongation alpha factor; c, enolase; d, alcohol dehydrogenase; e, fructose biphosphate aldolase; f, glyceraldehyde-3-phosphate dehydrogenase. **(B)** Quantification of protein intensities performed using ImageJ software.

The proteins bound to melatonin were purified at different time points during alcoholic fermentation (Figure 3). The detection of proteins bound to melatonin was inversely related with its intracellular concentration. No melatonin-binding proteins were detected during the first 8 h of fermentation (the lag phase of yeast growth; Figure 1), in which the first peak of intracellular melatonin was observed. Instead, coinciding with low levels of melatonin in the intracellular medium, six melatonin-binding protein bands appeared, presenting a stronger intensity at 20 h. Then, a clear decrease in the protein band intensity was observed, coinciding with the appearance of melatonin in the extracellular medium and with a small increase in intracellular melatonin. Once again, the decrease in intracellular melatonin overlapped with an increase in the protein band intensity at 30 h, which corresponded with the entrance in the stationary phase. During this phase, only very light bands were observed. Given that melatonin is a hormone produced in the pineal gland in humans (Lerner et al., 1958) and a key regulator of human chronobiological and endocrine functions (Jahanban-Esfahlan et al., 2018), this interaction may indicate the role of melatonin as a signal molecule in yeast.

Mass spectrometry analysis allowed us to identify these melatonin-binding proteins. The results, shown in Table 1, were obtained from different independent experiments. Surprisingly, most of the proteins identified participate in the glycolytic pathway: pyruvate kinase 1 (Pyk2p; band a), enolase (Eno1p, Eno2p; band c), fructose biphosphate aldolase (Fba1p; band d), and glyceraldehyde-3-phosphate dehydrogenase (Tdh1p, Tdh2p, Tdh3p; band f). The interaction of melatonin with all these proteins could suggest the presence of a glycolytic complex. Brandina et al. (2006) demonstrated that all glycolytic enzymes were associated with mitochondria in yeast, providing evidence for the formation of a macromolecular complex involving enolase and other glycolytic enzymes bound to the mitochondrial surface. The formation of this glycolytic complex, the glycolytic metabolon, could allow and regulate the channeling of different substrates into the mitochondria, such as pyruvate toward the TCA cycle or t-RNA mitochondrial import (Brandina et al., 2006; Graham et al., 2007). In *Arabidopsis thaliana*, glycolytic enzymes have also been described to be present on the surface of the mitochondria, in addition to the cytosol, forming a complex that supports substrate channeling to the mitochondria (Graham et al., 2007). In addition, Giegé et al. (2003) observed that seven out of 10 glycolytic enzymes of *Arabidopsis* cells were colocalized in the mitochondria.

**Table 1:** Identification of proteins purified from the SDS-PAGE gel using nanoLC-(Orbitrap) MS/MS.

Protein gel band	Gene name	Molecular weight (kDa)	Protein description (SGD database)	Sequence coverage, % number of peptides recovered by NanoLC-MS/MS	Mascot score
A	*PYK2*	54.5	Piruvate kinase 1	69.40	2048.70
B	*TEF1*	50.0	Translation elongation factor	56.55	1457.92
C	*ENO1*	46.8	Enolase	61.56	3294.52
	*ENO2*	46.9	Enolase	56.75	3343.08
D	*FBA1*	39.6	Fructose biphosphate aldolase	60.72	1551.78
E	*ADH1*	36.8	Alcohol dehydrogenase	56.32	797.83
F	*TDH1*	35.7	Triose-phosphate dehydrogenase	91.27	5885.33
	*TDH2*	35.8	Triose-phosphate dehydrogenase	74.40	4731.88
	*TDH3*	35.7	Triose-phosphate dehydrogenase	82.83	4219.04

Moreover, we identified an RNA-binding protein (Figure 3, band b), the cytoplasmatic translation elongation factor TEF1-A (Tef1p). Tef1p has also been reported to be part of the enolase complex in the mitochondria of *S. cerevisiae* (Brandina et al., 2006). Given that melatonin seems to be synthesized in the mitochondria (He et al., 2016), where it also develops its antioxidant function by preventing ROS toxicity (Acuña-Castroviejo et al., 2001; Okatani et al., 2002), melatonin may also act as a signal to activate the binding of the glycolytic complex. In addition, Cui et al. (2018) demonstrated that exogenous melatonin significantly altered the expression of glycolytic proteins, including enolase, fructose biphosphate aldolase and glyceraldehyde-3-phosphate dehydrogenase in wheat. Additionally, glyceraldehyde-3-phosphate dehydrogenase has been reported to be regulated on a daily basis by the circadian clock in *Neurospora*, an ascomycete fungus (Shinohara et al., 1998).

Finally, alcohol dehydrogenase was also detected but with the lowest Mascot score (Figure 3, band “e” and Table 1). Giegé et al. (2003) detected low activity of this enzyme in mitochondrial extracts and hypothesized that a low proportion of alcohol dehydrogenase could be associated with mitochondria.

## Conclusions

Melatonin is produced in the lag phase of yeast growth and is exported to the extracellular media during the stationary phase in fermentation conditions. Between intracellular and extracellular concentration peaks, melatonin seems to be bound to several proteins in *S. cerevisiae*. Most purified proteins participate in the glycolytic pathway, suggesting a possible complex of glycolytic enzymes bound to melatonin. This interaction may highlight the role of melatonin as a signal molecule in yeast. In addition, the interaction with TEF1-A could indicate that melatonin is part of the cell signaling process in stress conditions in yeast and that this glycolytic complex might facilitate the export of this molecule. Thus, this study reports for the first time the interaction of melatonin and proteins in yeast cells. Nevertheless, further studies are needed to understand the interaction between melatonin and yeast proteins to help elucidate the biological importance of melatonin in yeast.

## Data Availability Statement

All datasets generated for this study are included in the manuscript/supplementary files.

## Author Contributions

MM-P designed and carried out the experiments, analyzed, and discussed the results and wrote the manuscript. MV designed and carried out the experiments and participated in the discussion of the results. GB, AM, and M-JT designed the experiments, discussed the results, and revised the manuscript.

### Conflict of Interest

The authors declare that the research was conducted in the absence of any commercial or financial relationships that could be construed as a potential conflict of interest.

## Footnotes

^1^https://imagej.nih.gov/ij/
